# ASMT Regulates Tumor Metastasis Through the Circadian Clock System in Triple-Negative Breast Cancer

**DOI:** 10.3389/fonc.2020.537247

**Published:** 2020-10-21

**Authors:** FenFen Xie, LiLi Wang, YaJing Liu, ZhenBang Liu, ZuoYang Zhang, Jing Pei, ZhengSheng Wu, MuXin Zhai, YunXia Cao

**Affiliations:** ^1^Reproductive Medicine Center, Department of Obstetrics and Gynecology, The First Affiliated Hospital of Anhui Medical University, Hefei, China; ^2^NHC Key Laboratory of Study on Abnormal Gametes and Reproductive Tract (Anhui Medical University), Hefei, China; ^3^Key Laboratory of Population Health Across Life Cycle (Anhui Medical University), Ministry of Education of the People’s Republic of China, Hefei, China; ^4^Anhui Province Key Laboratory of Reproductive Health and Genetics, Hefei, China; ^5^Biopreservation and Artificial Organs, Anhui Provincial Engineering Research Center, Anhui Medical University, Hefei, China; ^6^Department of Histology and Embryology, Anhui Medical University, Hefei, China; ^7^School of Biomedical Sciences, The University of Queensland, Brisbane, QLD, Australia; ^8^School of Life Sciences, University of Science and Technology of China, Hefei, China; ^9^Department of Pathology, Anhui Medical University, Hefei, China; ^10^Department of Breast Surgery, The First Affiliated Hospital of Anhui Medical University, Hefei, China; ^11^First Clinical Medical College, Anhui Medical University, Hefei, China

**Keywords:** circadian clock system, acetylserotonin methyltransferase, triple negative breast cancer, migration, invasion

## Abstract

**Objective:**

Triple-negative (PR^−^, ER^−^, HER-2^−^) breast cancer (TNBC) is regarded as more aggressive and more likely to recur after medical care. Emerging evidence has demonstrated that the circadian clock system regulates cell-signaling pathways critical to cancer cell proliferation, survival and metastasis, meaning that it could be a good candidate for TNBC treatment. As such, the aim of the current study was to examine the molecular mechanism by which the circadian clock system contributes to cancer progression in TNBC.

**Methods:**

Cancer cells and primary breast cancer tissues were immunostained for the measurement of circadian clock proteins (CLOCK, BMAL1 and PER1) and acetylserotonin methyltransferase (ASMT). The association between ASMT and clock proteins was assessed using siRNA and Western blot. Transwell assays were used to detect cancer cell migration and invasion while MTT assays were utilized to evaluate cell proliferation.

**Results:**

Circadian clock proteins (CLOCK, BMAL1, and PER1) and ASMT expression were higher in TNBC and triple positive breast cancer (TPBC) compared with para-carcinoma tissues (PCTs). Intriguingly, there was an obvious correlation between circadian clock proteins and ASMT expression in both TPBC and TNBC. Similarly, circadian clock proteins and ASMT were expressed to a greater extent in BT-474 (triple-positive) cells than in MDA-MB-231 (triple-negative) cells. The inhibition of ASMT reduced circadian clock protein levels in both breast cancer cell lines. Further analysis showed that the expression levels of ASMT and circadian clock proteins did not correlate with clinical parameters such as age, tumor size, histologic grade and CK5/6, but increased significantly with lymphatic invasion in TNBC. In agreement with this finding, knockdown of ASMT significantly leads to reductions in migration and invasion in MDA-MB-231 cells. However, over-expression of CLOCK reversed the decreases seen in ASMT inhibited cells.

**Conclusion:**

Our study suggests that ASMT regulates the circadian clock system in breast cancer and inhibition of ASMT reduces the invasiveness of triple-negative breast cancer cells by downregulating clock protein in a certain extent, indicating the potential value of ASMT as a drug target for TNBC treatment.

## Introduction

Circadian rhythms are 24-h biological cycles controlled by a circadian system that is organized in a hierarchical manner. The circadian system master clock, located in the suprachiasmatic nucleus (SCN) of the hypothalamus, synchronizes the peripheral oscillators to ensure temporally coordinated physiology. Guided by the activity of the SCN, numerous hormones and genes are entrained to 24-h rhythms of expression and activity to ensure proper cellular function. Among these circadian-regulated hormones is melatonin (MT), which is considered to be an important linkage-molecule between the SCN and the peripheral biological clocks, and has been shown to fluctuate in accordance with environmental light (1). At the molecular level, the intracellular rhythm oscillator is a transcriptional/translation feedback loop generated by a set of interplaying clock proteins including three period proteins (PER1, PER2, and PER3), two cryptochromes (CRY1 and CRY2), CLOCK, and two BMAL proteins (2).

It is generally believed that the pineal gland is the major source of MT production, and MT plays important roles in many physiological and pathological functions, such as the regulation of circadian rhythms (3), antioxidative protection (4), tumor inhibition (5), and immunoregulation (6). The biosynthetic pathway of pineal MT occurs through hydroxylation, decarboxylation, acetylation and a methylation starting with L-tryptophan. Specifically, L-tryptophan is hydroxylated by tryptophan hydroxylase to produce 5-HT, which is then catalyzed by the serotonin N-acetyltransferase (NAT) to N-acetyl-5-hydroxytryptamine, which is ultimately catalytically converted by hydroxyndole-O-methyltransferase (HIOMT, also known as acetylserotonin ethyltransferase, ASMT) to MT (7). MT suppresses cancer cell proliferation (8) and thus, is suggested to benefit breast cancer patients undergoing radiation therapy (9).

Breast cancer, one of the most common types of cancer, is classified into different subtypes based on the presence of estrogen receptor (ER), progesterone receptor (PR) and growth factor receptor epidermal 2 (HER-2/neu). Accounting for nearly 15%–20% of all breast cancers, TNBC is characterized by aggressive behavior, poor prognosis, poor differentiation, visceral metastatic spread and an increased rate of node invasion (10). Compared with treatment options for other subtypes, treatment options for TNBC are very limited, as targeted therapies and endocrine therapies appear to be invalid due to the absence of ER, PR, and HER-2. Expression of MT receptors in TNBC in African American and Caucasian patients has been associated with overall survival (11). Overexpression of ASMT is indicated as a marker of pineal parenchymal tumors in the brain (12). However, the function of ASMT in TNBC is rarely addressed.

A reduction in the expression of clock proteins has been identified in MT receptor-deficient mice, which implies the regulation of circadian clock genes by melatonin (13). Although the disruption of circadian clock gene expression correlates with tumorigenesis in breast cancer (14), it has also been reported that repression of circadian machinery could improve anticancer efficacy by targeting cancer autophagy and metabolism (15).

Moreover, BMAL1 regulates pyruvate metabolism in MDA-MB-231 cells, and inhibition of BMAL1 decreases lung tumor nodules in triple-negative breast cancer mice fed a low-fat diet (16). To investigate the regulation between ASMT and clock in TNBC, the expression of ASMT and circadian clock proteins in breast cell lines and in TNBC patients was investigated systematically in our study. The association between ASMT and circadian clock proteins in breast cancer was also explored. Our results indicate that ASMT may regulate tumor invasion through circadian clock system in TNBC patients.

## Materials and Methods

### Subjects

Samples of 55 breast cancer patients (30 TPBC, 25 TNBC) were obtained from the First Affiliated Hospital of Anhui Medical University. Ethics approval was obtained from the ethics committee of clinical medical research, First Affiliated Hospital of Anhui Medical University and all patients signed informed consent prior to sample collection. The population statistics of TPBC and TNBC patients are summarized in **Table 1**. The detailed information of receptors expression is presented in **Supplementary Table 1**.

**Table 1:** Correlations between ASMT expression and clinical parameters in TPBC and TNBC patients.

Clinical parameters	TPBC	TNBC	*r*	ASMT Expression		*P*
				TPBC	TNBC	
**n**	30	25				
**Age**						
** <35**	0	2	0.138	0	13.96 ± 1.02	
** ≥35 <50**	12	11		21.88 ± 4.44	8.30 ± 2.95	**
** ≥50**	18	12		16.67 ± 7.83	6.17 ± 2.92	**
**Tumor type**						
** IDCA**	29	25	-0.099	20.60 ± 6.35	7.32 ± 2.81	**
** ILC**	1	0		20.81	0	
**Tumor size**						
** 1.0 to <2.0**	9	11	0.117	21.67 ± 5.32	6.91 ± 3.33	**
** 2.0 to <5.0**	21	14		19.29 ± 6.97	7.50 ± 2.74	**
**Histologic grade**						
** I**	1	0	-0.101	19.03	0	
** II**	26	15		20.00 ± 6.70	7.60 ± 3.13	**
** III**	3	10		19.70 ± 5.69	7.40 ± 2.17	**
**Lymphatic invasion**						
** (+)**	15	12	-0.217	20.75 ± 7.19	9.17 ± 2.89	**
** (-)**	15	13		19.14 ± 5.80	5.62 ± 1.26	**
**CK5/6**						
** (+)**	0	21	0.637	0	7.57 ± 2.99	
** (-)**	30	4		20.00 ± 6.52	6.00 ± 0.82	**

r: Correlation Coefficients.

The correlation analysis was performed by Spearman correlation test, **p < 0.01. The data were presented as mean ± S.E.M.

### Cell Culture

MCF-10A, BT-474, and MDA-MB-231 cells obtained from the American Type Culture Collection were maintained in DMEM (Thermo Fisher Scientific) supplemented with 10% fetal calf serum (Thermo Fisher Scientific), 55 µg/ml sodium pyruvate, 4 mg/ml glucose, 2 mM L-glutamine, 25 mM HEPES, 0.1 mg/L streptomycin, and 100 units/ml penicillin G (Sigma-Aldrich) in a 100 mm culture dish at a density of 3.0 × 10^5^ cells/ml. Cells were cultured in a humidified 5% CO_2_ incubator at 37°C. All cell lines used for experiments were between 8–12 passages.

### Immunohistochemistry Staining

The TPBC and TNBC sections were hydrated and treated with 1.5% hydrogen peroxide for 1 h at 37°C, followed by treatment in 0.05 M citrate-buffered saline (pH 6.0) at 95°C for antigen retrieval. The TPBC and TNBC sections were then incubated in 5% goat serum (Gibco) for 1 h at 37°C prior to incubation with the primary antibodies (1:200 dilutions) anti-BMAL1, anti-PER1, anti-ASMT (Abcam), anti-CLOCK (Abcam, 1:1000), and anti-serotonin N-acetyltransferase N-terminal (Sigma-Aldrich, 1:200) for 1 h at 37°C and overnight at 4°C. Subsequently, the TPBC and TNBC sections were incubated with the biotinylated goat anti-rabbit IgG (Santa Cruze Biotechnology, 1:200) for 1 h at 37°C, followed by incubation with avidin-biotin peroxidase complex (1:200) and dehydration with ethanol. Data are presented as the percentages of positive areas of ASMT, NAT, CLOCK, BMAL1, and PER1 relative to the total area. Six uncontinuous fields were selected from one slide at a magnification of 200×.

### Western Blotting

The protocols used for the preparation of the cell lysate and Western blotting have been described previously (17). Briefly, total protein was extracted from cells using prechilled lysis buffer (50 mM Tris/HCl, pH 8.0, 150 mM NaCl, 0.1% SDS, 1% Nonidet p-40, 0.5% sodium deoxycholate, and a mixture of protease inhibitors). The protein samples were resolved on 15% SDS-PAGE gels and then transferred onto nitrocellulose membranes. Blots were probed with anti-CLOCK antibody (1:5000), anti-BMAL1 antibody (1:1000), anti-PER1 antibody (1:1000), anti-ASMT antibody (1:1000) and anti-NAT antibody (1:2000) overnight at 4°C, followed by horseradish peroxidase-conjugated secondary antibodies, and were visualized using an ECL chemiluminescence detection kit (Amersham Life Sciences). Quantification was performed by densitometry using ImageJ software.

### Transfection of DNA and Small Interfering RNA

Cancer cells were seeded in 6-well plates at 2 × 10^5^ cells/well. On the next day, 5 μl of Lipofectamine 3000 (Thermo Fisher Scientific) was mixed with 200 μl of Opti-MEM (Thermo Fisher Scientific) and 2.5 μl of siRNA (5’-GGCAUUCUGGUAAUUGAAATT-3’) or (5’-CCCACUUAUGUGUGACCUUTT-3’) (20 μM) (Shanghai Jima), and 5 μg of plasmid DNA was mixed with 200 μl Opti-MEM, and the mixtures were added to each well. Twenty-four hours following transfection, the medium was replaced with 1 ml of complete RPMI-1640 medium. Cells were transfected for a total time of 2 days prior to analysis.

### MTT Assay

MDA-MB-231 cells were seeded in 96-well plates at a density of 10^4^ cells/well and maintained overnight. At the indicated times, 10 μl of 5 mg/ml MTT (3-[4,5-dimethylthiazol-2-yl]-2,5-diphenyl tetrazolium bromide) solution (BestBio, Shanghai) was added to each well, and the cells were incubated for 4 h at 37°C. To dissolve the formazan crystals, 150 μl of DMSO was added per well prior to absorbance measurement at 490 nm using a spectrophotometer.

### Migration and Invasion Assay

Cell migration and invasion abilities were measured using Corning Transwell insert chambers. For the invasion assay, the chamber was first coated with 1 mg/ml Matrigel for 4 h at 37°C. Then, MDA-MB-231 cells were seeded onto the upper chamber at 2 × 10^5^/ml in a total volume of 100 μl of serum-free medium. Meanwhile, 800 μl of RPMI medium supplemented with 10% FBS was added to the lower chamber. After 48 h of incubation at 37°C and 5% CO_2_, cells that had migrated to the lower surface of the upper chamber were fixed with 4% paraformaldehyde, stained with 0.5% crystal violet, and counted under a microscope.

### Statistical Analysis

Statistical data were analyzed using ANOVA and Dunnett’s multiple comparisons test, which were conducted with SPSS 24.0 software. The correlation analysis was performed by the Pearson’s correlation coefficient. Values are expressed as the mean ± S.E.M. Statistical significance was defined as *p* < 0.05.

## Results

### Correlation of ASMT With the Circadian Clock System in Breast Cancer

**Figure 1:**
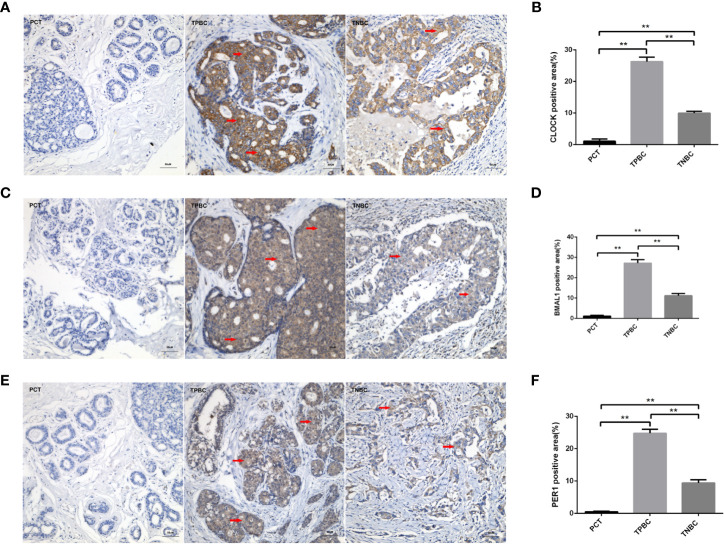
Higher expression levels of the circadian clock proteins in TPBC compared with TNBC and para-carcinoma tissues (PCT). Immunostaining analysis using antibodies against CLOCK **(A)**, BMAL1 **(C)**, and PER1 **(E)** on tumor sections and PCT. The nuclei were stained with Hematoxylin (blue). Red arrows were used to indicate the positive area. **(B, D, F)** Quantification of CLOCK, BMAL1 and PER1 positive areas relative to total areas. Data are the mean ± SEM (TPBC,n=30; TNBC, n=25; PCT, n=8). **p < 0.01.

**Figure 2:**
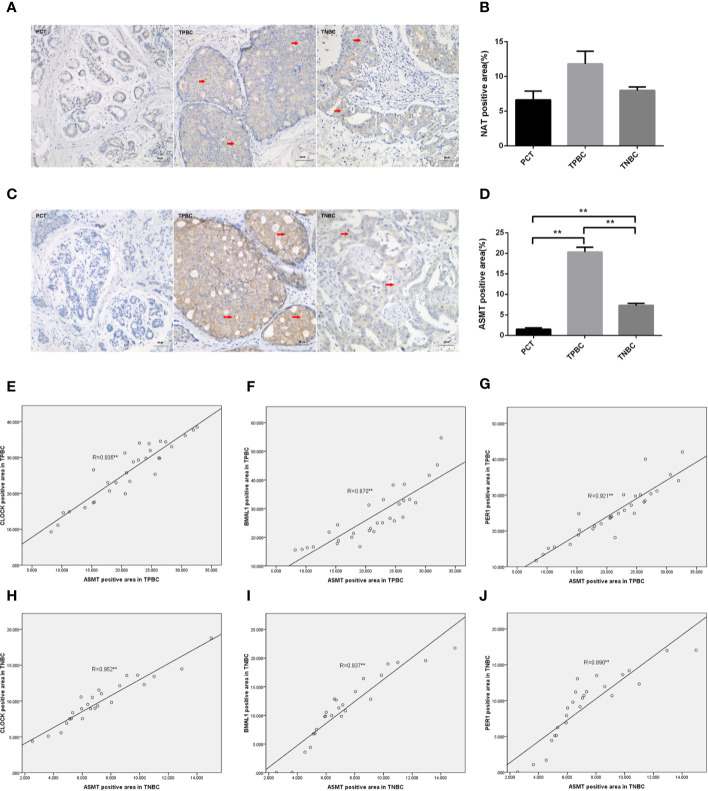
Correlations between ASMT and the circadian clock system in TPBC and TNBC patients. Immunostaining analysis using antibodies against NAT **(A)** and ASMT **(C)** on tumor sections and PCT. The nuclei were stained with Hematoxylin (blue). Red arrows were used to indicate the positive area. **(B, D)** Quantification of NAT and ASMT positive areas relative to total areas. Data are the mean ± SEM (TPBC,n = 30;TNBC,n=25;PCT,n=8). **p < 0.01. **(E–G)** Correlations between ASMT expression and CLOCK, BMAL1, and PER1 expressions in TPBC. **(H–J)** Correlations between ASMT expression and CLOCK, BMAL1, and PER1 expressions in TNBC. The correlation analysis was performed by the Pearson’s correlation test, **p < 0.01.

To determine the exact action of the circadian clock system in TNBC, the expression levels of CLOCK, BMAL1 and PER1 were first examined by immunohistochemistry. TPBC and para-carcinoma tissues (PCTs) were chosen as controls for a better understanding of circadian clock system function. There were significant increases in the expression levels of CLOCK (**Figures 1A, B**), BMAL1 (**Figures 1C, D**), and PER1 (**Figures 1E, F**) in TPBC (n=30) and TNBC (n=25) compared to those in PCTs (n=8). Interestingly, the expression levels of these proteins were decreased in TNBC compared to those in TPBC. Our results also showed that ASMT but not NAT expression was higher in TPBC and TNBC patients than in PCTs (**Figures 2A–D**). Similarly, a reduction in ASMT expression was found in TNBC compared that in TPBC. Studies have found that ASMT, but not NAT, is the limiting enzyme in the generation of MT (18). Therefore, the relationship between ASMT and the circadian clock system was further assessed. Not surprisingly, decreased ASMT expression was correlated with decreased CLOCK (**Figure 2E**), BMAL1 (**Figure 2F**), and PER1 (**Figure 2G**) expression in both TPBC and TNBC patients (**Figures 2H–J**).

### Regulation of Circadian Clock Systems by ASMT in Breast Cancer Cell Lines

**Figure 3:**
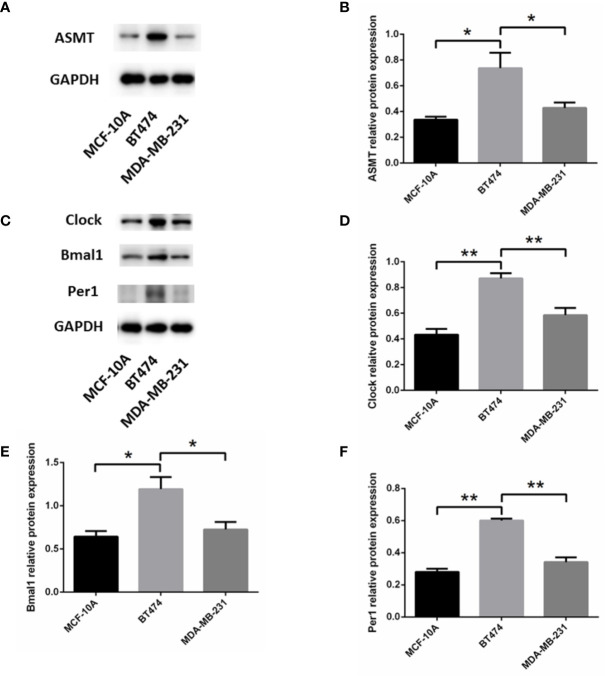
Expression of ASMT and the circadian clock proteins increases in BT-474 compared with MDA-MB-231 and MCF-10A cells. **(A, B)** ASMT expression measured by Western blotting and quantification of ASMT level relative to GAPDH. **(C–F)** CLOCK, BMAL1 and PER1 expressions measured by Western blotting and quantification of the circadian clock system levels relative to GAPDH. Data are the mean ± SEM (n = 3). *p < 0.05, **p < 0.01.

To determine the mechanism that links ASMT with the circadian clock system, triple-positive or triple-negative breast cell lines were utilized for further study. MCF-10A cells, which express very low amounts of ER, PR, and HER-2 (19), were used as a control for MDA-MB-231 cells. The expression levels of ASMT and circadian clock system were tested by Western blotting. As shown in **Figure 3**, ASMT, CLOCK, BMAL1, and PER1 expression levels were decreased in MCF-10A and MDA-MB-231 cells compared with those in BT-474 cells, but no significant differences were found between MCF-10A and MDA-MB-231 cells. Consistently, it has been demonstrated that the ER signaling pathway leads to the upregulation of the circadian clock system in breast cancer cells (20). Taken together, these results showed that breast cancer cell lines are good models to investigate the regulation between ASMT and the circadian clock system.

**Figure 4:**
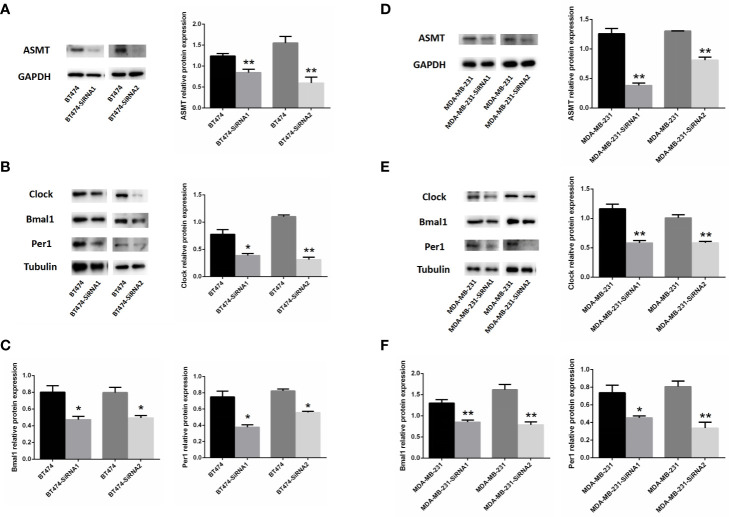
Inhibition of ASMT reduces the circadian clock system levels in BT-474 and MDA-MB-231 cells. **(A)** ASMT expression measured by Western blotting following siRNA treatment and quantification of ASMT levels relative to GAPDH in BT-474 cell. **(B, C)** The expression and quantification of CLOCK, BMAL1, and PER1 protein relative to tubulin measured by Western blotting in BT-474 cell. Data are the mean ± SEM (n = 3). *p < 0.05, **p < 0.01. **(D)** ASMT expression measured by Western blotting following siRNA treatment and quantification of ASMT levels relative to GAPDH in MDA-MB-231 cell. **(E, F)** CLOCK, BMAL1 and PER1 expression and quantification relative to tubulin measured by Western blotting in MDA-MB-231 cell. Data are the mean ± SEM (n = 3). *p < 0.05, **p < 0.01.

As such, BT-474 (**Figure 4A**) and MDA-MB-231 (**Figure 4D**) cells were treated with 2 types of siRNAs targeting different regions of ASMT to exclude potential off-target effects. Both siRNA treatments significantly reduced ASMT expression to very low levels. Noticeably, the expression levels of CLOCK, BMAL1, and PER1 were reduced significantly after the loss of ASMT (**Figures 4B, C, E, F**). These results further implied that the reduction in the circadian clock system seen in BT-474 and MDA-MB-231 cells might be due to downregulation of ASMT.

### ASMT and Circadian Clock System Expression Correlate Positively With Lymphatic Invasion in TNBC

**Figure 5:**
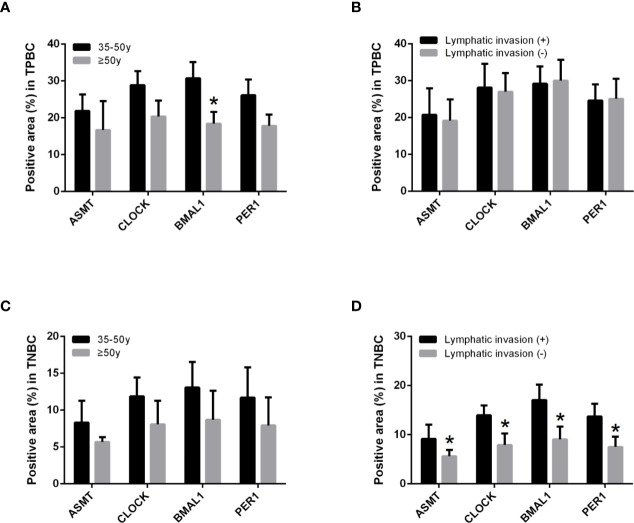
ASMT expression and the circadian clock system expressions correlate positively with lymphatic invasion in TNBC. **(A)** Only BMAL1 expression levels decreased in the elder TPBC. **(B)** Expression levels of ASMT and the circadian clock proteins do not change with lymphatic invasion in TPBC. **(C)** Expression levels of ASMT and the circadian clock proteins do not change with age in TNBC. **(D)** Expression levels of ASMT and the circadian clock proteins are higher with lymphatic invasion in TNBC patients. Data are the mean ± SEM (TPBC, n = 30; TNBC, n = 25). *p < 0.05.

To better identify the characteristics of breast cancer patients according to ASMT expression and clock expression, their correlations with clinicopathological factors such as age and lymphatic invasion were further analyzed in the TPBC and TNBC groups. The expression levels of ASMT and circadian clock proteins increased significantly with lymphatic invasion in TNBC but not in TPBC (**Figures 5B, D**). However, the correlations with age were not significant in either group (**Figures 5A, C**). In summary, these findings indicate that ASMT and the circadian clock system may promote lymphatic invasion in TNBC.

### Inhibition of ASMT Leads to Reductions in Migration and Invasion by Downregulating Clock Expression in MDA-MB-231 Cells

**Figure 6:**
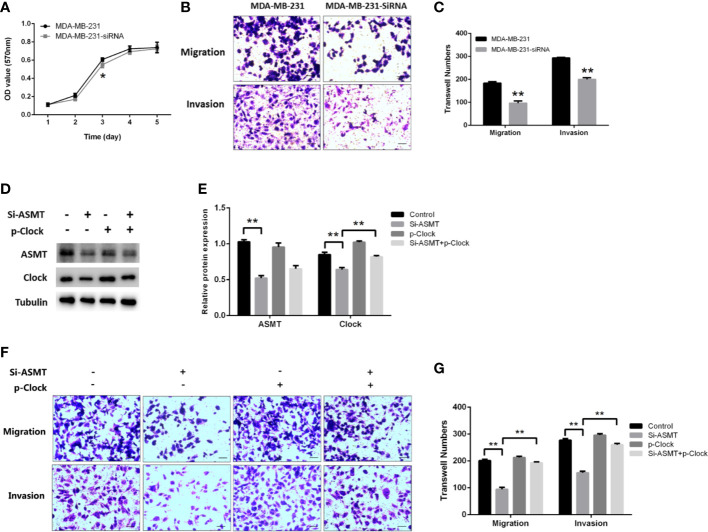
Inhibition of ASMT leads to reductions in migration and invasion by downregulating clock expression in MDA-MB-231 cells. **(A)** Cell proliferation following the loss of ASMT expression as measured by MTT assay. Data are the mean ± SEM (n=8). **(B, C)** Representative images and counts of cells migration and invasion following ASMT-SiRNA transfection. Cells were fixed with 4% paraformaldehyde, stained with 0.5% crystal violet, and counted under microscope. Scale bar=100μm. Data are the mean ± SEM (n=8). **(D, E)** Expressions and quantifications of ASMT and CLOCK measured by Western blotting. Tubulin was used as an internal control for protein quantification. Data are the mean ± SEM (n=3). **(F, G)** Representative images and counts of cells migration and invasion following ASMT-SiRNA and CLOCK plasmid DNA cotransfection. Scale bar = 100 µm. Data are the mean ± SEM (n = 8). **p < 0.01.

Since the expression of ASMT appeared to be altered with lymphatic invasion in TNBC, we assessed the role of ASMT in MDA-MB-231 cells. As measured by MTT assay, the inhibition of ASMT had little effect on cell proliferation, except for a statistically significant difference on the 3^rd^ day (**Figure 6A**). In agreement with the clinical data, a decrease in invasion was found following ASMT inhibition. Furthermore, there was also a reduction in cell migration after the loss of ASMT (**Figures 6B, C**). The effects of ASMT siRNA and CLOCK plasmid transfection were verified by Western blot (**Figures 6D, E**). Surprisingly, the over-expression of CLOCK led to no significant changes in migration and invasion compared to control, suggesting clock is not the only factor that regulates the migration and invasion of MDA-MB-231 cells. (**Figures 6F, G**). Nevertheless, the results demonstrate that inhibition of ASMT reduce the invasiveness of triple-negative breast cancer cells by downregulating clock protein in a certain extent.

## Discussion

Currently, there is no universal treatment for TNBC due to the lack of valid therapeutic targets. Thus, it is beneficial to identify new targets that could be used for TNBC therapy. In the present study, we showed that ASMT regulates the circadian clock system, which may reveal a new mechanism for cancer cell invasion and migration in TNBC.

Recent research has shown that the circadian clock system plays a crucial role in tumorigenesis. For instance, an increase in BMAL1 expression was found in malignant pleural mesothelioma (21). In contrast, Tang et al. revealed that BMAL1 acts as a tumor suppressor in tongue squamous cell carcinoma (22). It has also been shown that PER1 expression decreases significantly in breast tumors (23). In agreement with previous study that showed breast tumor tissues exhibited higher CLOCK expression than healthy breast tissues (24), circadian clock system expression was found to be increased in breast cancer patients compared to that in PCTs, which suggests that the circadian clock system could be a tumor promoter rather than a suppressor. We also found that the expression of circadian clock proteins was greater in TPBC than in TNBC. Consistently, circadian clock proteins were expressed at higher levels in BT-474 cells than in MDA-MB-231 cells. ER-alpha regulates the transcription of CLOCK (20). HER-2 promotes the activation of multiple mitogen-activated protein kinase pathways that regulate cell migration and proliferation (25). The inhibition of ERK signaling was found to block Bmal1 expression (26). To date, however, there is no underlying mechanism that explains the data presented here.

The expression and activity of NAT could be controlled by circadian clock genes (27). However, studies on the modulation of ASMT by circadian clock genes are still lacking. It has been shown that both ASMT and NAT are suppressors of the mutated CLOCK gene (28). Nevertheless, our data demonstrate a strong correlation between ASMT and circadian clock genes (CLOCK, BMAL1, PER1). This result is consistent with a study that showed the same change pattern of mRNA expression for the CLOCK, PER1, CRY1, CRY2, and ASMT genes following pinealectomy (29). Moreover, our data strongly support the correlations of the circadian clock system with ASMT in breast cancer tissues. Most importantly, our data showed direct regulation of the circadian clock system by ASMT. How ASMT modulates the circadian clock system remains unknown. Although melatonin could be involved in this regulation, another factor should be taken into account in further studies since ASMT also catalyzes the conversion of 5-hydroxy-indoleacetate to 5-methoxy-indoleacetate, a reaction in the tryptophan metabolism pathway (30).

The alteration of circadian rhythms correlates with increased susceptibility to breast cancer in humans (31). In the current study, elevated expression of ASMT and circadian clock proteins with lymphatic invasion was found in TNBC. Similarly, a previous study suggested an association of clock expression with deeper depth of invasion in colorectal cancer (32). Our data showed that knockdown of ASMT in triple-negative breast cancer cells significantly reduced cell migration and invasion, but this phenotype was reversed when CLOCK was over-expressed simultaneously. Recently, Wang et al. demonstrated that BMAL1 facilitates breast cancer cell invasiveness through upregulation of MMP9 expression at themRNA and protein levels (33). Intriguingly, BMAL1 dimerizes with CLOCK, which enhances the histone acetyl transferase activity of CLOCK (34). Although the underlying mechanism involving ASMT, CLOCK, and tumor invasion still remains to be elucidated, we demonstrate that inhibition of ASMT leads to reductions in the invasiveness of TNBC cells by downregulating clock expression in a certain extent.

In summary, the current study clearly illustrates that ASMT regulates the circadian clock system in breast cancer and that ASMT and clock proteins are overexpressed in breast cancer patients. The downregulation of ASMT in TNBC may reduce cancer cell migration and invasion by inhibiting clock expression, providing a new molecular mechanism that regulates the invasiveness of TNBC. Therefore, ASMT and clock could be effective drug targets, especially in TNBC patients with metastasis.

## Data Availability Statement

The raw data supporting the conclusions of this article will be made available by the authors, without undue reservation.

## Ethics Statement

The studies involving human participants were reviewed and approved by Ethics committee of clinical medical research, First Affiliated Hospital of Anhui Medical University. The patients/participants provided their written informed consent to participate in this study.

## Author Contributions

FX and LW contributed equally to this work. YC and YL were responsible for the conceptualization and supervision of the study. FX performed the experiments, analyzed the data and constructed the tables and figures. LW and YL designed experiments and interpreted the data. ZL, ZZ, JP, ZW, and MZ were responsible for technical and material support. LW and FX wrote the original manuscript, which was edited by YC and YL. All authors contributed to the article and approved the submitted version.

## Funding

The present work was supported by the National Natural Science Foundation of China (81771653, 81871216), Non-profit Central Research Institute Fund of Chinese Academy of Medical Sciences (2019PT310002), the National key research and development program of China (2016YFC1000204, 2017YFC1001300), the Key Science Research Project at universities of Anhui Province (KJ2017A194), the Excellent Young Talents Support Program at universities of Anhui Province (2009SQRZ046), and the Key Excellent Young Talents Support Program at universities of Anhui Province (gxyqZD2017031).

## Conflict of Interest

The authors declare that the research was conducted in the absence of any commercial or financial relationships that could be construed as a potential conflict of interest.
