# Supplementary material for: ASMT Regulates Tumor Metastasis Through the Circadian Clock System in Triple-Negative Breast Cancer
Source: Front Oncol. 2020 Oct 21;10:537247. doi: 10.3389/fonc.2020.537247 (PMC7609885; doi:10.3389/fonc.2020.537247)
Supplement: Supplementary file 1 [file Table_1.docx]

**Suppl. Table 1 The status of ER/PR and HER2 expression of breast cancer tissues in TPBC patients**

| Serial number | ER | | PR | | HER2 | | |
| --- | --- | --- | --- | --- | --- | --- | --- |
| 1  2  3  4  5  6  7  8  9  10  11  12  13  14  15  16  17  18  19  20  21  22  23  24  25  26  27  28  29  30 | | **+**  **+**  **+**  **+**  **+++**  **+++**  **++~+++**  **++~+++**  **+++**  **++**  **+++**  **+++**  **++**  **+++**  **++~+++**  **++~+++**  **+++**  **++**  **+++**  **+++**  **+++**  **+++**  **+++**  **+++**  **++**  **+++**  **++**  **+++**  **++**  **+++** | | **+**  **+**  **+**  **+**  **+++**  **+++**  **++~+++**  **++~+++**  **+++**  **+++**  **+++**  **++**  **++**  **++**  **++~+++**  **++~+++**  **+++**  **+++**  **++**  **+++**  **+++**  **++**  **+++**  **+++**  **++**  **+++**  **+++**  **+++**  **++**  **++** | | **+++**  **+++**  **+++**  **+++**  **+++**  **+++**  **+++**  **+++**  **+++**  **+++**  **+++**  **+++**  **+++**  **+++**  **+++**  **+++**  **+++**  **+++**  **+++**  **+++**  **+++**  **+++**  **+++**  **+++**  **+++**  **+++**  **+++**  **+++**  **+++**  **+++** |  |

**+: The proportion of positive cells was less than 20%**

**++: The proportion of positive cells was between 20% to 80%**

**+++: The proportion of positive cells was more than 80%**

**There are no expression of ER/PR and HER2 in breast cancer tissues of TNBC patients.**
